# Deployment of vaccine cold chain equipment in resource-limited settings: lessons from the Gavi Cold Chain Optimization Platform in Cameroon

**DOI:** 10.1093/inthealth/ihae010

**Published:** 2024-02-09

**Authors:** Jude Nkwain, Vouking Marius Zambou, Sangwe Clovis Nchinjoh, Valirie Ndip Agbor, Amani Adidja, Clarence Mbanga, Nnang Nadege Edwidge, Shalom Tchokfe Ndoula, Andreas Ateke Njoh, Demba Diack, Pietro Di Mattei, Owens Wiwa, Ousmane Diaby, Yauba Saidu

**Affiliations:** Gavi, the Vaccine Alliance, Chem. du Pommier 40, 1218 Le Grand-Saconnex, Geneva, Switzerland; United Nations Children's Fund (UNICEF), Country Office, Yaoundé, 335 Rue 1810, Cameroon; Clinton Health Access Initiative Inc., Yaoundé, P.O. Box 2664, Cameroon; Clinical Trial Service Unit and Epidemiological Studies Unit (CTSU), Nuffield Department of Population Health, University of Oxford, Oxford, OX3 7LF, UK; Faculty of Medicine and Biomedical Sciences, University of Yaoundé 1, Yaoundé, P.O. Box 1364, Cameroon; Clinton Health Access Initiative Inc., Yaoundé, P.O. Box 2664, Cameroon; Clinton Health Access Initiative Inc., Yaoundé, P.O. Box 2664, Cameroon; School of Global Health and Bioethics, Euclid University, Bangui, P.O. Box 157, Central African Republic; School of Global Health and Bioethics, Euclid University, Bangui, P.O. Box 157, Central African Republic; Gavi, the Vaccine Alliance, Chem. du Pommier 40, 1218 Le Grand-Saconnex, Geneva, Switzerland; Gavi, the Vaccine Alliance, Chem. du Pommier 40, 1218 Le Grand-Saconnex, Geneva, Switzerland; Clinton Health Access Initiative Inc., Yaoundé, P.O. Box 2664, Cameroon; Department of Studies and Projects, Ministry of Public Health, Yaoundé, P.O. Box 1937, Cameroon; Clinton Health Access Initiative Inc., Yaoundé, P.O. Box 2664, Cameroon

**Keywords:** Cameroon, cold chain equipment optimization, deployment, vaccine cold chain, vaccines

## Abstract

**Background:**

Lack of or use of suboptimal cold chain equipment (CCE) is a major barrier to optimal immunization coverage and equity. Gavi established the CCE optimization platform (CCEOP) in 2015 to help eligible countries modernize their cold chain systems. However, there are limited data on CCE deployment at country level. We present lessons learnt from deploying CCE from the Gavi CCEOP in Cameroon.

**Methods:**

This cross-sectional study collected data on the number of days items of CCE spent at each point on their trajectory from the entry port to 62 randomly selected health facilities in Cameroon.

**Results:**

Once equipment arrived at the entry port, it took 10 d for customs clearance, 2 d from customs clearance to warehousing and 257 d (>9 mo) from the warehouse to facilities. Upon arrival at the facilities, it took a median of 53 (range 0–395) d from installation to final commissioning: most of the days (median=210) were spent between installation and final commissioning. The major causes of delays included insufficient coordination and communication across all levels, poor documentation and final commissioning.

**Conclusion:**

Early engagement on customs clearance, strengthening coordination and communication, ensuring proper documentation, as well as eliminating final commissioning, could significantly improve implementation of the program.

## Introduction

Immunization is widely acknowledged as one of the most successful and cost-effective public health interventions in history. Despite this, many national immunization programs fail to meet their annual coverage and equity goals, which results in 25 million unimmunized and 5 million underimmunized children each year.^1^ One key underlying reason for this failure is the inequity in the availability and distribution of vaccine cold chain equipment (CCE), which is the cornerstone of any immunization program.^2^ Indeed, published reports suggest that a lack of adequate numbers of quality CCE items is one of the major determinants of low vaccination coverage and equity, and a key impediment to the introduction of new vaccines into public health systems in many low- and middle-income countries (LMICs).^3^

These devices are critical in ensuring a continuous supply of quality vaccines at service delivery points of an immunization program.^4–6^ However, CCE systems of many immunization programs in LMICs are not optimal to handle the increasing number of new vaccines, putting huge quantities of vaccines at risk.^7^ This challenge, coupled with inadequate temperature monitoring and maintenance systems, could compromise vaccine potency, and affect the quality of immunization shots administered to children or lead to massive financial loss from vaccine wastage.^8^ A national cold chain inventory in Cameroon showed that nearly one-half of all facilities across the country had no cold chain storage capacity, and 93% of CCE items were non-functional or suboptimal.^9^

To mitigate these risks, Gavi, the Vaccine Alliance, established the CCE optimization platform (CCEOP) in 2015 to support governments in modernizing their cold chain systems infrastructure so as to better safeguard antigens and expand their cold chain networks to previously unequipped areas.^10^ Through joint financial investment, participating countries gain access to optimal (WHO prequalified) cold chain storage technologies and benefit from innovative procurement strategies for obtaining these technologies. By doing so, Gavi-eligible countries would be able to leverage sufficient and optimal CCE to improve on their immunization coverage and equity, reducing under-five mortality from vaccine-preventable diseases. Cameroon applied and was approved for the CCEOP in 2016. The project scope, from upstream procurement to downstream immunization service delivery, was to span >3 y of equipment deployments totaling 1383 solar direct drive refrigerators (SDDs), 1700 electric ice-lined refrigerators (ILRs), four freezers and equipment accessories.

Although the implications of frail cold chains have been reported in many studies,^5,11,12^ publications on the deployment of CCE in resource-limited settings are scarce. In a bid to start addressing this scarcity, we share our experiences of deploying devices from the CCEOP in Cameroon, with the objective of guiding future cold chain optimization interventions in resource-limited settings. Specifically, we sought to describe: the national-level timeliness of CCE procurement, deployment, installation and coordination; and facility-level equipment deployment timeliness, quality and functionality.

## Materials and Methods

### Study design and setting

This was a cross-sectional study conducted from May to August 2020 and it involved districts and health facilities in Cameroon that had received refrigerators or freezers and accessories (automatic voltage stabilizers [AVSs], fridge tags and lightning rods) from Year 1 CCEOP deployments. Eligible for inclusion in the study were district vaccine stores and health facilities across all 10 regions of Cameroon located in the 33 health districts where UNICEF, Clinton Health Access Initiative (CHAI), Inc. or other partners have piloted or conducted interventions to address operational feasibility related to security concerns and areas of extensive remoteness with extreme difficulties in access. Additionally, vaccine cold chain management staff at the selected facilities were eligible for inclusion. We excluded 138 facilities that had already received postinstallation supervision from the National Logistics Working Group to avoid duplication of efforts.

### Sampling

Of the 381 facilities eligible for inclusion in this study, a sample size of 62 facilities (16.3%) was initially targeted through simple random sampling without replacement after stratification by region. The final list of selected facilities was reviewed to ensure operational feasibility, with purposeful replacement of a few inaccessible or non-functional facilities.

### Data collection and procedure

Data were collected at central and facility levels. At health facility level, quantitative data were collected using an observation checklist, which covered elements on the quality of CCE installation, the availability of associated equipment such as voltage stabilizers and fridge tags, equipment functionality, as well as the timeliness of equipment delivery, installation and commissioning. Field staff visited the selected health facilities and, with the assistance of the facility staff, inspected the various items on the checklist. For each item, the field staff indicated Yes/No for availability and functionality of items and assessed whether equipment met the predefined quality criteria (Table 1). In addition, field staff assessed documentation available at the health facility to determine timelines for delivery, installation and commissioning. At the central level, a desk review of records was used to collect information on the dates of information on shipping, customs clearance, distribution, installation and commissioning of CCE following 12 time points:

1. Customs exoneration application: that is, the date on which Cameroon initiates the process for obtaining clearance of customs duties.
2. Customs exoneration obtained: date that the customs exoneration is granted by the Ministry of Finance.
3. Customs clearing agent contracted.
4. Batch shipment arrival at port in Cameroon.
5. Batch customs clearance in Douala.
6. Batch transport to warehouse: the date on which the CCEOP equipment batch is transported to central warehouse for interim storage.
7. Start date of dispatch from warehouse to facility.
8. End date of dispatch from warehouse to facility.
9. Start date of installations.
10. End date of installations.
11. Start date of final commissioning (30 d postinstallation).
12. End date of final commissioning (30 d postinstallation).

**Table 1.** Equipment installation quality and functionality grading

Item	Grading
**Quality of equipment installation**	
**Electric fridges/freezers**	
Plug	Marked as meeting quality criterion if the device plug is connected properly directly into the wall outlet (that fitting is consistent with Cameroon sockets) and with no loose connections
Power	Marked as meeting quality criterion if the operation bottom of the device is turned on, cables neat and organized
Air circulation	Marked as meeting quality criterion if at least 30 cm is left between the wall and the device for proper air flow
Voltage regulator	Marked as meeting quality criterion if the device is not cut off or damaged, with indicator lights turned on and supplying voltage to the device
Level	Marked as meeting quality criterion if equipment is not slanted or wobbly
**Solar fridges**	
Solar panel	Marked as meeting quality criterion if solar panels are installed in a location with plenty of sunlight, not angled toward shade and are free of excessive dust
Cables	Marked as meeting quality criterion if indoor cables are neatly clipped and organized, and if outdoor solar cables are properly concealed and routed into the facility using electrical conduits for protection
Earth rod/lightning rod	Marked as meeting quality criterion if the rod is correctly fastened to the ground with sheathed cables protected from view
Air circulation	Marked as meeting quality criterion if at least 30 cm is left between the wall and the device for proper air flow
Level	Marked as meeting quality criterion if equipment is not slanted or wobbly
**Functionality and equipment performance**	
Refrigerators	Marked as functional if vaccines were stocked in the equipment
Freezers	Marked as functional if the indicator light was illuminated
Temperature-monitoring devices (fridge tags)	Marked as functional if the battery lights indicated not weak or dead
Automatic voltage stabilizers	Marked as functional if the indicator lights were illuminated

### Data analysis

Data were analyzed using Microsoft Excel (2016, Microsoft Corporation, Redmond, WA, USA) and R programming software version 4.3.0 (2023, The R Foundation for Statistical Computing, Vienna, Austria). The analysis employed descriptive statistics comprising mean, frequency and proportion. Timeliness of deployment (customs clearance, delivery, installation and commissioning) was measured by referencing dates listed on CCE delivery notes, installation checklists and final commissioning documents. Using the same approach as central timeliness indicators, average durations for each of these steps were then summed to calculate an overall average duration of facility-level distribution processes (delivery, installation and commissioning). The cumulative timeliness indicator was calculated by adding the timeliness indicators for individual deployment ‘steps’ for which there were dates provided. Equipment installation quality and functionality were determined based on the criteria in Table 1.

## Results

Because central-level time points had missing data, only two (13%) of the 12 time points had dates available. As such, a mix of dates at the facility and service providers were used for this study. This was done by soliciting responses from the service providers regarding the dates when items were shipped to health facilities, installed and commissioned. The date of documentation available with the service provider reached 60% (nine of the 12 dates). The date provided for the customs exoneration application was retained in the full analysis. Where the service provider reported different dates from those obtained at health facility level, for batches one and two of the CCEOP equipment deployment, averages of both batches were used in the analysis.

Seventy-one items of CCE (70 fridges and one freezer) were delivered across the 60 surveyed sites, which consisted of 48 health facilities and 12 district vaccine stores. Out of the 60 sites that received CCEOP equipment, 57% received iced line refrigerator (ILR) devices and the rest (43%) received SDDs. Most sites (85%) received only one refrigerator, while nine sites (15%) received two to three ILR or SDD devices.

### Timelines for deployment, installation and commissioning of CCE

**Figure 1.**
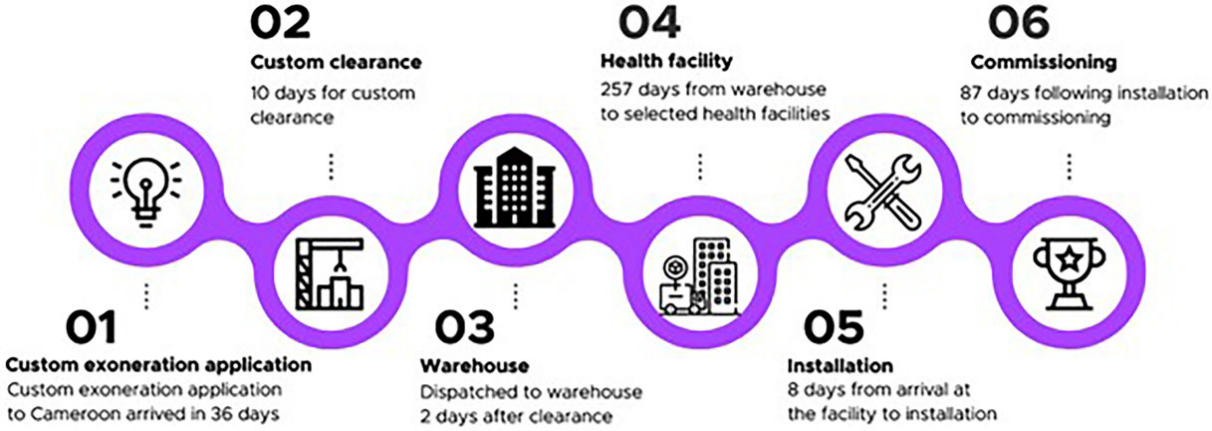
Timeline of key activities in CCE deployment, installation and commissioning.

Figure 1 shows the timelines for CCE deployment, namely, custom clearance, dispatch to the warehouse, distribution to health facilities, installation and commissioning. Overall, the total time from the CCEOP customs exoneration application to the end of CCE dispatch to facilities equaled 305 d (>10 mo). Nearly 9 mo of this time was spent dispatching both batches of CCE from the central warehouse to the facilities. Additionally, Figure 1 shows the breakdown of duration by each step of the central deployment process, from the date of the CCE customs exoneration application on 7 January 2019, to the last date of dispatch of the CCE warehouse to facilities on 10 December 2019. While it took 36 d from applying for customs exoneration to obtaining a customs exoneration certificate, on average, both batches of equipment were custom-cleared within 10 d and dispatched to the warehouse 2 d after clearance. Dispatching of CCE from the warehouse to the facilities took nearly 9 mo (257 d).

Upon arrival at the facilities, it took a median of 53 (range 0–395) d for installation and final commissioning. The largest portion of this time (median=210 d) was spent between installation and final commissioning. CCE arrival at the facility to installation was much shorter, averaging 8 d in total (min=0, max=53). The South (n=4) and Southwest (n=6) regions had same-day installation of equipment upon arrival, while the West (mean=33 d, n=5) and Adamaoua (mean=16 d, n=2) regions had the longest duration from arrival to installation.

Only 56% of assessed CCE had delivery notes with clearly written dates. Therefore, a large proportion of missing data means timeliness outcomes were unavailable for a large portion of the sample.

### CCE installation

#### CCE and accessories delivered and installed

**Figure 2.**
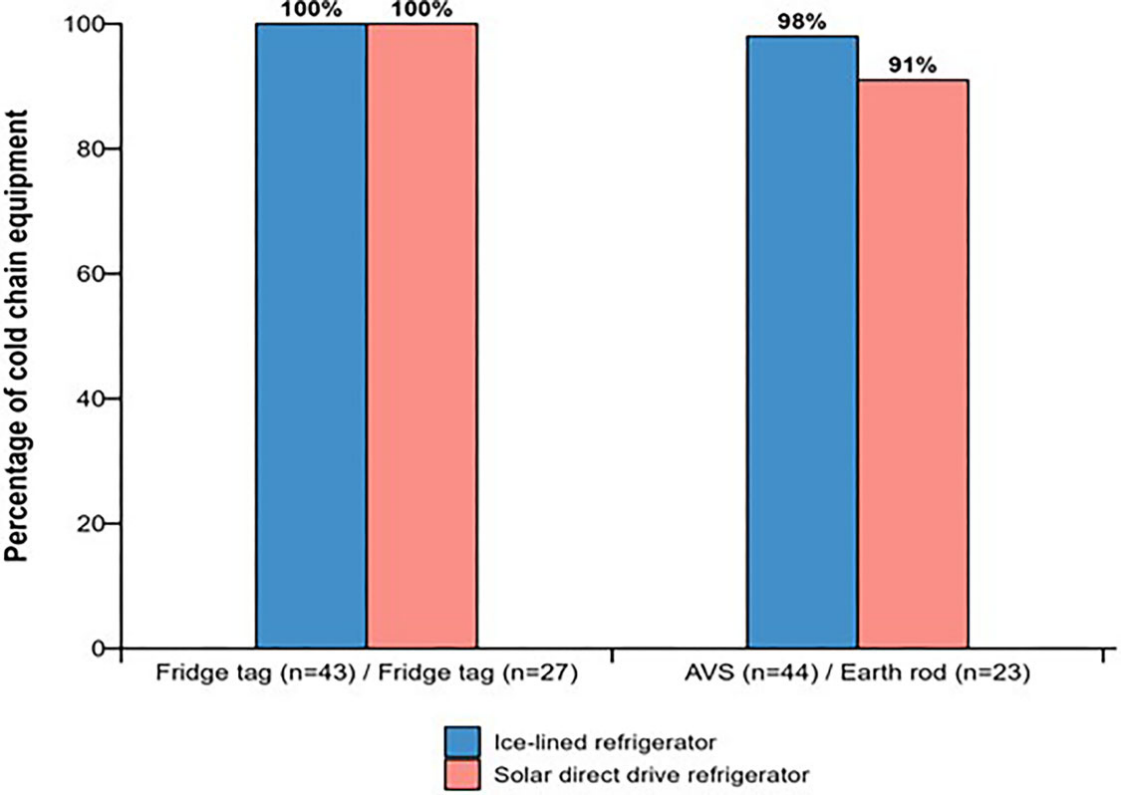
Proportion of ILR and SDD accessories installed out of those delivered.

All ILR CCE was delivered with the appropriate equipment. In other words, all 43 refrigerators were delivered with temperature-monitoring devices and AVSs, and the freezer was also delivered with an AVS. As shown in Figure 2, only 89% of SDDs (24/27) were delivered with all relevant accessories; all were delivered with a temperature-monitoring device, but only 24 out of 27 (89%) were delivered with an earth rod. In addition, there were ILRs (11%) marked as mistakenly having received earth rods, although the study staff were not able to confirm or refute this.

Most of the accessories that were delivered with the CCE were installed. Overall, 98% of ILR CCE (43/44) had the AVS installed, while 91% of the 23 SDD CCE had the lightning (earth) rods installed upon delivery. All (70/70) ILR and SDD CCE (excluding the freezer) had the temperature-monitoring devices installed with the equipment.

#### Quality and functioning of installed CCE accessories

Table 2 shows the quality of CCE accessories installed at facilities. Only 33% (n=9) of SDDs had properly trunked and protected cables, while 70% (n=19) of SDDs had earth rods correctly fastened to the ground with sheathed cables protecting them from view. Nevertheless, all CCE was found to have been properly leveled with the floor (27 SDDs and 44 ILRs), with all SDDs having adequate space for air circulation.

### Quality of CCE installation

For both ILR and SDD CCE, the component that had the lowest quality score when observing installation quality were cables. Only 68% (n=30) of ILR CCE met this criterion, with only 33% (n=9) of SDDs having properly trunked and protected cables at the time of the survey (Table 2). Similarly, 70% of SDDs (n=19) had earth rods correctly fastened to the ground with sheathed cables protecting them from view. Regarding equipment placing, all CCE items were found to have been properly leveled with the floor (27 SDDs and 44 ILRs), with 100% of SSDs also having adequate space allowing air circulation. Assessment of ILR quality showed that the proportions achieved were a bit lower, with 89% having proper air circulation (n=39), 84% (n=37) with the standard device plug properly secured and functioning, and 82% (n=36) with a functioning and properly installed voltage stabilizer.

**Table 2.** Quality and functioning of CCE and accessories installed at facilities

CCE/accessory	Frequency	Percentage (%)
**ILR CCE functioning (N=44)**		
Secured plugs	37	84.1
Power cables	30	68.2
Air circulation	39	88.6
Voltage stabilizer	36	81.8
Level with floor	44	100.0
**Percentage of SDD CCE meeting quality criteria (N=27)**		
Solar panels not obstructed	25	93
Neatly trunked cables	9	33
Concealed trunked earth rod	19	70
Proper air circulation	27	100
Correct level with the floor	27	100

### Equipment functionality after installation

**Figure 3.**
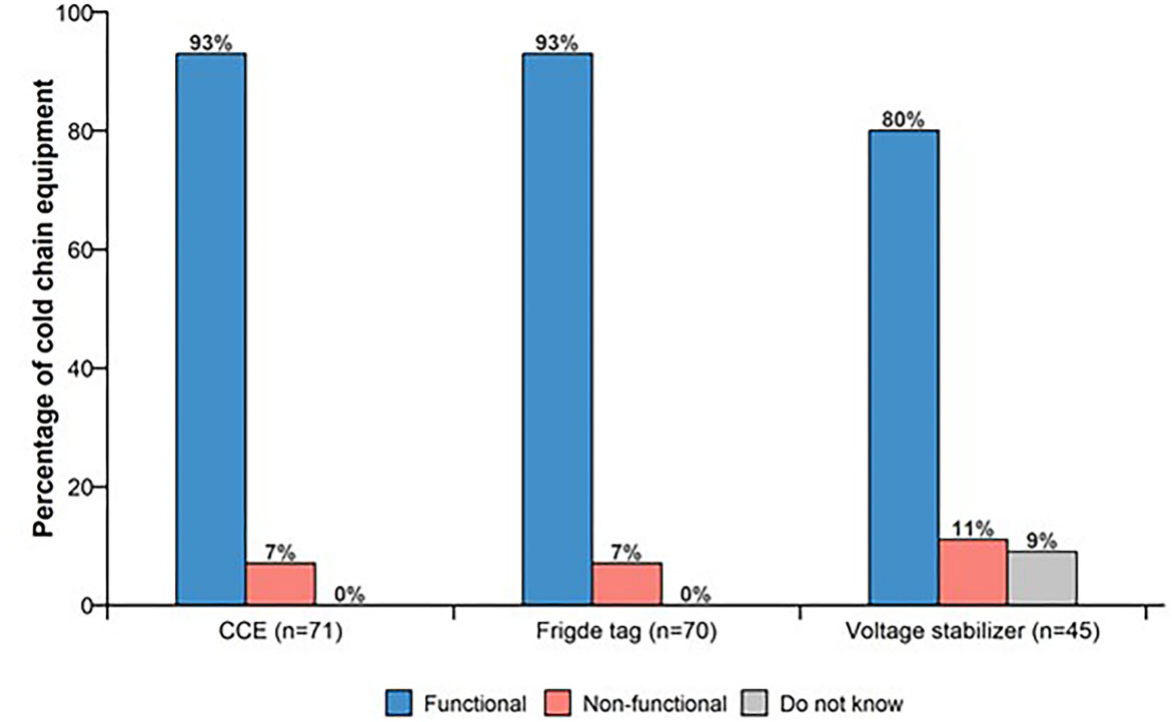
Functionality of CCE, fridge tags and voltage stabilizers.

The functionality of the installed CCE at the time of the survey is presented in Figure 3. Almost all of the CCE (93%) was found to be functional; however, one freezer located at a district vaccine store, and four refrigerators at facilities (three ILRs and one SDD), were not functional at the time of the survey. Also, 93% (65 out of 70) of the temperature-monitoring devices were functional at the time of the survey. The lowest proportion of functionality was evident with AVSs, for which 80% (36 of the 45 voltage stabilizers received) were functional.

## Discussion

This study aimed to describe the national-level timeliness of CCE distribution and installation, as well as facility-level equipment timeliness, quality and functionality. We found that it took a total of 364 d from customs clearance to the commissioning of CCE. To speed up CCE deployment, the timelines for the distribution, installation and commissioning of equipment were scheduled to occur with overlap. Based on the central-level plans, CCE installation was scheduled to commence on 23 February 2019, and commissioning was planned to be completed as early as May 2019. However, there seems to have been a lag between the initial dispatch of CCE and arrival at facilities when comparing central plans with facility-level documentation, as the first documented arrival date was 25 April 2019. This is >2 mo later than the planned central timeline of 23 February 2019. In addition, final commissioning (30 d postinstallation) took more than three times longer than expected. While the deployment process took about 12 mo, customs clearance was completed in just 10 d. One of the enabling factors for this short timeline was the close follow-up by the Project Management Team (PMT). This led to early customs exoneration application, which facilitated the obtention of the custom exoneration certificate prior to equipment arrival. However, despite the shorter time taken for customs clearance, significant delays were observed in the other phases of deployment.

These delays could be attributed to the bureaucracies and systematic structure of the health system management and leadership. While the PMT was established and functional, program structures required multiple approvals and actions by different stakeholders at central, regional and district levels, which may have led to stalemate and inaction at some levels. For instance, distribution to private facilities was halted due to delays with an official letter from the government attributing CCE to private health facilities. About 70% of target health facilities consisted of private health facilities; this delay, which lasted several months, could partly explain why distribution to health facilities took longer than initially planned. Another aspect that could have contributed to delays was a lack of coordination at subnational level, as in some instances health facility staff were not adequately informed about the arrival of CCE, which led to delays with the official reception of CCE at health facilities. Some of these were compounded by the lack of a telecommunications network at some sites. This finding is congruent with the postulations of Di Giulio et al. 2010 that poor coordination strategies among multiple implementing agencies and stakeholders in developing countries could cause breakdowns and delays in program delivery, bureaucracy and budget overruns.^13^ The costs of these delays can be detrimental to the success of the program and achieving its target goals, considering that delays and cost overruns can raise the capital-output ratio, thus decreasing the efficacy of the investment.^14^

To address some of the underlying causes of delays, Fos et al. 2022 suggests several considerations to strengthen partnership and coordination in public health systems. First, coordination must be inclusive, bringing together all government departments and levels, as well as partners like development organizations, academic institutions, local authorities and civil society. Second, structural elements of coordination bodies are crucial, such as the availability of coordination structures and regular meetings, the importance of leveraging already existing coordination mechanisms, as well as clear roles, mandates and sufficient authority. Third, organizations in charge of coordination need to have sufficient resources, including finance, communication and other tools such as terms of reference and standard operating procedures. Finally, strong political leadership and collaboration incentives encourage effective coordination.^15^

Our study also showed that most facilities did not document when equipment and accessories were received, installed and commissioned. This is not in line with 2021 recommendations by WHO and UNICEF that warrant actors in the various tiers of the vaccine cold chain optimization program to institute, and adhere to, appropriate documentation and authorization practices.^16^ Adherence to best documentation practices is critical to improve logistics information, as well as facilitate future interventions to improve cold chain management, including cold chain inventory updates across various districts and health facilities. The finding that many facilities had no system in place to track equipment highlights the need for continuous in-service training of health workers on documentation and its use in the management of logistics to enhance the effectiveness of the vaccine cold chain system.^17^

Additionally, most CCE and accessories were installed and functional at the time of the survey. However, a significant proportion of the voltage stabilizers were non functional. This is worrying as it has been noted that 59% of healthcare facilities in LMICs, including Cameroon, lack reliable electricity,^18^ highlighting the critical need for ensuring that AVSs are installed as CCE. Without these voltage stabilizers, vaccine integrity may be compromised during power outages, and in the long run, vaccine fridges and freezers are likely to be damaged in a short time.

## Study limitations

Challenges were faced at both central and facility levels in obtaining CCEOP deployment dates. Deployment dates began at application for customs exoneration all the way to facility-level delivery, installation and final commissioning of CCE. At the facility level, only about one-half of the 71 items of CCE included in the survey had delivery and installation documentation readily available with the dates clearly written (56% [n=40] and 58% [n=41], respectively). Equipment commissioning forms were even more scarce, with only 16% (n=11) of all the 71 items of CCE having documentation, including clearly written dates, available. With such small sample sizes, it was difficult to estimate with precision the duration of the deployment process at health facility levels for about one-half of the study sites.

### Conclusions

Our study showed that the implementation of the CCEOP program witnessed significant delays in deploying CCE against the shorter planned timelines. Early engagement on customs clearance preparations, including exoneration application and obtention of customs exoneration certificate, considerably reduced customs clearance timelines. However, insufficient coordination and communication across stakeholders at central, regional and district health services, poor documentation practices, significant delays between installation and commissioning, as well as non-functional accessories, such as voltage stabilizers, could hamper successful implementation of this program in similar contexts. Strengthening coordination and communication mechanisms with stakeholders across all levels, disseminating and ensuring guidance on proper documentation is adhered to, as well as early start and close follow-up for the customs clearance process, could significantly improve implementation of the program. In addition, protocols of final commissioning CCE 30 d after installation and before use should be avoided, as this has the unfortunate consequence of creating undue delays, directly impacting the urgency with which the program should be implemented.

## Data Availability

The data underlying this article will be shared on reasonable request to the corresponding author.
