# Erratum to: deployment of vaccine cold chain equipment in resource-limited settings: lessons from the Gavi Cold Chain Optimization Platform in Cameroon

**DOI:** 10.1093/inthealth/ihae027

**Published:** 2024-03-12

This is an erratum to: Jude Nkwain, Vouking Marius Zambou, Sangwe Clovis Nchinjoh, Valirie Ndip Agbor, Amani Adidja, Clarence Mbanga, Nnang Nadege Edwidge, Shalom Tchokfe Ndoula, Andreas Ateke Njoh, Demba Diack, Pietro Di Mattei, Owens Wiwa, Ousmane Diaby, Yauba Saidu, Deployment of vaccine cold chain equipment in resource-limited settings: lessons from the Gavi Cold Chain Optimization Platform in Cameroon, *International Health*, 2024; https://doi.org/10.1093/inthealth/ihae010.

In the original publication of this manuscript, the given and last names of the ninth co-author were transposed. This should read: “Andreas Ateke Njoh.”

Under **Results** (in the body of the paper), the first sentence should read: “Because central-level time points had missing data, only two (13%) of the 12 time points had dates available.” instead of: “Because central-level time points had missing data, only two (13%) of the 15 time points had dates available.” And the fourth sentence should read: “The date of documentation available with the service provider reached 60% (nine of the 12 dates).” instead of: “The date of documentation available with the service provider reached 60% (nine of the 15 dates).”

Under **Discussion**, in the fifth paragraph, the second sentence should read: “However, a significant proportion of the voltage stabilizers were non functional.” instead of: “However, a significant proportion of the voltage stabilizers were not installed.”

The emendations have been made to the article.

